# Integrating machine learning for the optimization of polyacrylamide/alginate hydrogel

**DOI:** 10.1093/rb/rbae109

**Published:** 2024-09-02

**Authors:** Shaohua Xu, Xun Chen, Si Wang, Zhiwei Chen, Penghui Pan, Qiaoling Huang

**Affiliations:** Research Institute for Biomimetics and Soft Matter, Fujian Provincial Key Laboratory for Soft Functional Materials Research, Department of Physics, College of Physical Science and Technology, Xiamen University, Xiamen 361005, China; Jiujiang Research Institute of Xiamen University, Jiujiang 332000, China; Tan Kah Kee Innovation Laboratory, Xiamen 361102, China; Research Institute for Biomimetics and Soft Matter, Fujian Provincial Key Laboratory for Soft Functional Materials Research, Department of Physics, College of Physical Science and Technology, Xiamen University, Xiamen 361005, China; Research Institute for Biomimetics and Soft Matter, Fujian Provincial Key Laboratory for Soft Functional Materials Research, Department of Physics, College of Physical Science and Technology, Xiamen University, Xiamen 361005, China; Jiujiang Research Institute of Xiamen University, Jiujiang 332000, China; Research Institute for Biomimetics and Soft Matter, Fujian Provincial Key Laboratory for Soft Functional Materials Research, Department of Physics, College of Physical Science and Technology, Xiamen University, Xiamen 361005, China; Jiujiang Research Institute of Xiamen University, Jiujiang 332000, China; Research Institute for Biomimetics and Soft Matter, Fujian Provincial Key Laboratory for Soft Functional Materials Research, Department of Physics, College of Physical Science and Technology, Xiamen University, Xiamen 361005, China; Jiujiang Research Institute of Xiamen University, Jiujiang 332000, China

**Keywords:** alginate/polyacrylamide hydrogel, Bayesian optimization, machine learning, flexible electronics, stretchability

## Abstract

Hydrogels are highly promising due to their soft texture and excellent biocompatibility. However, the designation and optimization of hydrogels involve numerous experimental parameters, posing challenges in achieving rapid optimization through conventional experimental methods. In this study, we leverage machine learning algorithms to optimize a dual-network hydrogel based on a blend of acrylamide (AM) and alginate, targeting applications in flexible electronics. By treating the concentrations of components as experimental parameters and utilizing five material properties as evaluation criteria, we conduct a comprehensive property assessment of the material using a linear weighting method. Subsequently, we design a series of experimental plans using the Bayesian optimization algorithm and validate them experimentally. Through iterative refinement, we optimize the experimental parameters, resulting in a hydrogel with superior overall properties, including heightened strain sensitivity and flexibility. Leveraging the available experimental data, we employ a classification algorithm to separate the cutoff data. The feature importance identified by the classification model highlights the pronounced impact of AM, ammonium persulfate, and *N*,*N*-methylene on the classification outcomes. Additionally, we develop a regression model and demonstrate its utility in predicting and analyzing the relationship between experimental parameters and hydrogel properties through experimental validation.

## Graphical abstract

**Figure:**
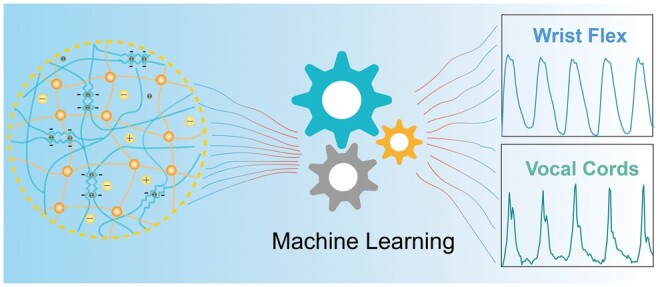

## Introduction

Hydrogels are swollen polymer materials with water as the dispersion media. The hydrogel has the capacity to absorb water within its polymer network, with absorption levels varying from over 10% to as high as 99% [1, 2]. Compared to rubbers, hydrogels are naturally soft, flexible, biocompatible with high and reversible elongation [3]. Herein, hydrogels are widely applied in many fields, including tissue engineering [4, 5], contact lenses [6], biosensors [7–10], drug delivery [11], artificial skin [12], soft electronics [13, 14] and wound dressing [15, 16].

Although hydrogel materials have been proven to have significant potential for various applications, traditional hydrogels still have many shortcomings, such as poor mechanical properties (brittleness and susceptibility to tearing under stress) and low conductivity, which limit their applications [17, 18]. In recent years, even though the development of material design and optimization are rapid [19], the design and optimization of hydrogels with desirable properties are still challenging. There are many experimental parameters involved in the fabrication of hydrogels, and the interplay among different experimental parameters complicate the optimization process. Herein, the traditional optimization approach using experiments and statistics requires a substantial human resources and material resources. Consequently, further development is required in the rational design and optimization of smart hydrogels for biosensing, to better realize the specific application.

Machine learning is a subset of artificial intelligence based on mathematics. It can create a mapping relationship between multi-variable input and output by training a model on available data, enabling the prediction of unknown results [20, 21]. It has been extensively applied in data analysis fields for uncovering potential patterns among datasets [22]. For supervised learning, a machine learning model is trained on a labelled dataset using a classification algorithm, enabling it to forecast discrete targets, such as unknown data categories in the parameter space [23]. Utilizing the regression algorithm, a regression model can be established to predict the target within the parameter space based on the relationship between the input and target parameters [24–26]. In addition, some algorithms designed for optimization process facilitate the efficient collection of data and mitigate resource overhead. The application of those algorithms can expedite the optimization process and further identify the optimal value more efficiently, with less experiments. For example, Tao *et al.* [30, 31] developed a synthetic self-driving platform for producing metal nanoparticles using Gryffin algorithm.

During the past few years, AI has been extensively employed in the development of hydrogels, including detecting, predicting and optimizing their properties, data processing and even discovering new materials for hydrogels [27–29, 32–34]. For example, Miao *et al.* [35] applied a deep neural network to analyze both the resistance and volume responses of hydrogels to assist with taste recognition. Wang *et al.* [36] introduced artificial neural networks to predict and optimize the swelling behavior of temperature-responsive hydrogels. Li *et al.* [37] developed a machine learning model to forecast the hydrogel-forming ability of nucleoside derivative, and discovered two rarely reported cation-independent nucleoside hydrogels. Zhang *et al*. [38] developed a fast, autonomous high-throughput method to characterize hydrogel rheological properties by integrating automated sensing with physics-guided machine learning.

Even though many studies have applied machine learning algorithms to optimize hydrogels, most focus on only one or two properties of the hydrogels. However, for the application of hydrogels, several properties need to meet the basic requirements. In this study, we focus on the five most important properties of hydrogels for better application in sensors, including strain sensitivity, elongation, fracture energy, hysteresis and resistivity. We first design and prepare a strain-sensing hydrogel for applications in bionics and flexible electronics. Then, we employ a Bayesian optimization algorithm to design a set of experimental plans to adjust experimental parameters to obtain hydrogels with improved properties. We use classification algorithms to filter out parameter combinations that lead to solutions incapable of forming hydrogels. Finally, we utilize regression algorithms for a more in-depth analysis of the relationship between experimental parameters and material properties.

## Experimental section/methods

### Fabrication of hydrogels

Acrylamide (AM) and disodium calcium ethylenediaminetetraacetate (EDTA-Ca) were dissolved in deionized water, and then *N*,*N*-methylenebisacrylamide (MBA), ammonium persulfate (APS) and sodium alginate (SA) powder were added and stirred. Let the solution rest until all the internal bubbles dissipated, then dissolve glucolactone (GDL), sodium chloride (NaCl) and tetramethylethylenediamine (TEMD) in deionized water. Stir the mixture of AM and SA, then gradually introduce the GDL, NaCl and TEMD mixture into the blended AM and SA solution using a syringe. The mixture was cast into a Teflon mod and covered with a glass slide before irradiating under a UV light (500 W) for 10 min.

### Characterizations

The hydrogels were frozen at −20°C and freeze-dried for 5 days. The dried samples were analyzed by a scanning electron microscope (Zeiss, Sima-HD-01-36) equipped with an EDS analyzer. Thermal weight loss behavior of the freeze-dried hydrogels was compared by a thermogravimetric analyzer (NETZSCH TG209F1, German) with a heating rate of 10 K/min under nitrogen flow at 70 ml/min. The molecular structures were studied by a Fourier transform infrared spectrometer manufactured by Thermo Fisher Scientific (Nicolet-iS10).

### Mechanical properties

The mechanical property tests involving elongation and fracture energy was controlled by a single arm testing machine (JHY-5000, Jingheyuan). The uniaxial tensile test was performed along *z*-axis on a cuboid hydrogel (15 mm × 8 mm × 2 mm) with a tensile speed of 100 mm/min. The maximum elongation at break was calculated as follow:

(1) $$\mathrm{Elongation}=\frac{\Delta L}{L_{0}}\times 100\%,$$

where ΔL and $L_{0}$ refer to the change in length at break and the original length of the sample for tensile deformation. The fracture energy was further calculated by integrating the area under the stress–strain curve.

(2) $$\mathrm{Fracture}\;\mathrm{energy}=S{\times L}_{0}\times W\times T,$$

where S refers to the area enclosed by the stress–strain curve and the coordinate axis. $L_{0}$, W and T refer to the original length, width and thickness of the sample.

Hysteresis effects were assessed through stretch cycle experiments on a rectangular-shaped sample (45 mm×15 mm×2 mm). The sample was stretched to a predetermined elongation of 200% and then unloaded to zero force, with a gauge length of 15 mm. The closed loop formed by the stretching curve is called a ‘hysteresis loop’. The hysteresis loop can be divided into upper (stretching) and lower (releasing) curves at the maximum strain point. The areas enclosed by these two curves and the coordinate axes are calculated and denoted as $S_{S}$ and $S_{R}$, respectively. And the hysteresis effects can be calculated by the following equation:

(3) $$\mathrm{Hysteresis}=\frac{S_{S}-S_{R}}{S_{S}}.$$

### Electrical property test

The electrical properties of the hydrogels were gauged using an impedance meter (TH2840B, Changzhou Tonghui Electronic Co., Ltd., China). To monitor the resistance, a copper electrode was pasted on the surface of the hydrogel sample, and linked to the impedance meter via a wire. The resistivity was further calculated by the following formula:

(4) $$\mathrm{Resistivity}=\frac{R_{0}\times W\times T}{L},$$

where $R_{0}\;$and L are the resistance and the length of the hydrogel. When hydrogel serves as a strain sensing material, the higher the strain sensitivity of the material, the greater the change in resistance under the same deformation. The relative change in resistance was determined by the formula: $\left(R-R_{0}\right)/R_{0}$, where R refers to the resistance of hydrogel under applied strain. The strain sensitivity is further calculated from the area ($S_{\mathrm{GF}}$) under the gauge factor (GF) curve:

(5) $$\mathrm{Strain}\;\mathrm{sensitivity}=\frac{S_{\mathrm{GF}}}{L_{\max}},$$

where $L_{\max}\;$is the maximum elongation length.

### Dataset

Experimental features for the Bayesian optimization process consisted of the concentrations of seven components: AM, SA, NaCl, MBA, APS, sodium calcium edetate (EDTA-Ca) and TEMED. The concentrations of these seven components were denoted as variables (features) $X=\left\{x_{1},\;x_{2},x_{3},x_{4},x_{5},x_{6},x_{7}\right\}$ and imported into the dataset. For each experimental set *X*, at least three replicates were fabricated and tested. We used the median value to represent the results of three samples made under the same experimental conditions.

The data label *Y* was computed from five properties of the hydrogels, including strain sensitivity, elongation, fracture energy, hysteresis and resistivity. For solving multi-objective evaluation and optimization problems, the linear weighting model is widely utilized, with the simple additive weighting method being a classic linear weighting algorithm. This approach, however, ignores variations in units and value ranges among different objectives. By assigning corresponding weights to each objective function and linearly weighting all objectives, a comprehensive utility function can represent the overall evaluation objective, thereby converting multi-objective problems into single-objective ones. To mitigate the impact of range and unit discrepancies among multiple targets, it is essential to normalize each target independently prior to applying linear weighting. The following equation shows the normalization process:

(6) $$f(x)=\frac{f_{i}^{\max}\left(x\right)-f_{i}(x)}{f_{i}^{\max}\left(x\right)-f_{i}^{\min}\left(x\right)},$$

where $f_{i}(x)$ denotes the *i*th target, $f_{i}^{\max}\left(x\right)$ and $f_{i}^{\min}\left(x\right)$ represent the maximum and minimum values of $f_{i}(x)$, respectively. Then, linear weighting was applied:

(7) minimizex⁡∑n=1∞ωifi'(x),

where fi'(x) refers to the *i*th objective function following normalization, with $\omega_{i}\;$signifies the weight assigned to the *i*th objective function, and $\sum_{i=1}^{n}\omega_{i}=1$.

After linearly weighting, the weight of each target property was computed using the entropy weight method provided by SPSSPRO. The label Y was then obtained by

(8) $$Y=-\;(0.27y_{1}+0.31y_{2}+0.17y_{3}-0.16y_{4}-0.09\;y_{5}),$$

where Y represents the comprehensive property of the hydrogel, and $y_{1}$, $y_{2}$, $y_{3}$, $y_{4}$ and $y_{5}$ represent the normalized values of strain sensitivity, elongation, fracture energy, hysteresis and resistivity. The smaller the Y value, the better the properties of the gel.

### Bayesian optimization process

The features and labels in the dataset were imported into Bayesian optimization algorithm, which further determined the experimental parameters for hydrogel fabrication. The GPyOpt library (https://sheffieldml.github.io/GPyOpt/) was used for the Bayesian optimization algorithm. Three acquisition types, expected improvement (EI), maximum probability of improvement (MPI) and lower confidence bound (LCB) were used. The hyperparameters used in the algorithm were all default values in the library without optimization. The main reason is that optimizing the hyperparameters would require a substantial amount of additional experimental data. Such optimization is more suitable for situations where large amounts of experimental data can be collected automatically in a short period of time. In contrast, data collection in this study relied on manual operation by the experimenter, which was time-consuming. The default values in the database are generally more universal. Therefore, considering the purpose of this study as well as time and cost constraints, we decided not to optimize the hyperparameters of the Bayesian optimization algorithm.

## Results and discussion

### Design of a double-network hydrogel based on polyacrylamide and sodium alginate

**Figure 1.**
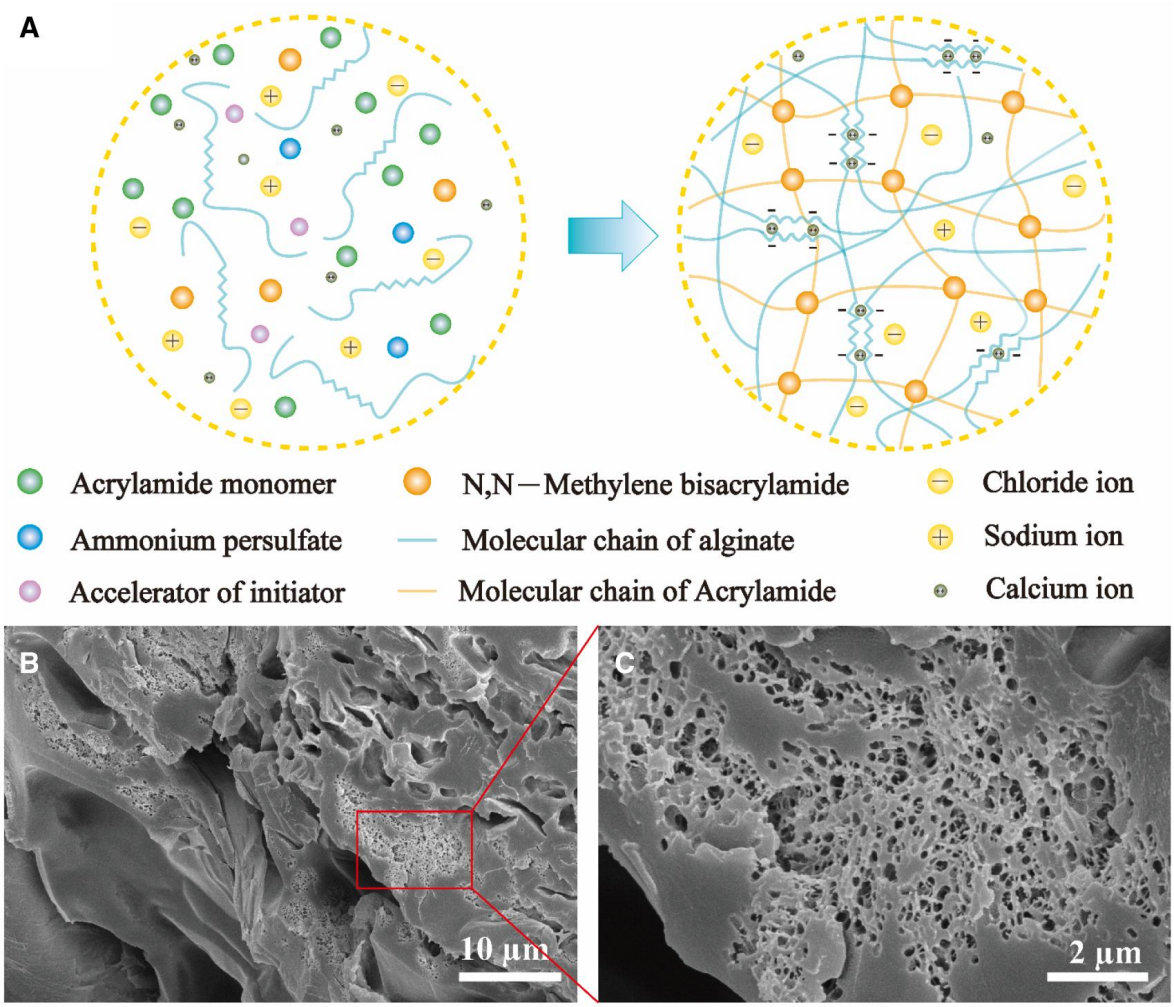
(**A**) Design principle of interpenetrating double network hydrogel based on polyacrylamide and alginate. (**B**, **C**) SEM images of hydrogels at different magnification.

Polyacrylamide/alginate-based interpenetrating double network hydrogels leverage the unique properties of both alginate and polyacrylamide, and exhibit exceptional mechanical strength compared to traditional hydrogels [39]. In this article, we implement corresponding adjustments derived from this dual-network hydrogel to optimize its overall properties (Figure 1). The alginate polymer chain crosslinks through calcium ions, whereas the formation of the polyacrylamide network is initiated by APS with MBA as the cross-linking agent. Calcium ions are introduced gradually through GDL and EDTA-Ca. GDL, a lipid, hydrolyzes in water, reducing the solution’s acidity. Under acidic conditions, the chelated calcium ions in EDTA-Ca are released as free calcium ions, which then serve as cross-linking points for alginate. We introduce NaCl into the hydrogel as an ionic conductor to improve the conductivity of the material, i.e. reducing the resistivity. Figure 1B and C represents the cross-sectional images of the freezing-dried polyacrylamide/alginate hydrogel. It is clear that the freeze-drying process generates a multi-scale porous three-dimensional structure (Figure 1B). In addition, the interpenetrating double network hydrogel displays a more intricate structure with numerous tiny holes (Figure 1C). It implies that the ice crystals formed during freezing are not uniform which influence the dimensions of the pores. Within a few experiments, we obtained hydrogels with a maximum elongation of 928% at fracture, a strain sensitivity of 8.71 and a fracture energy of 176 kJ/m^3^. A comparison of thermogravimetric analysis and infrared absorption spectra of each sample is illustrated in Supplementary Figures S1 and S2.

### Optimizing experimental parameters based on the Bayesian optimization algorithm

There are many parameters affecting gel properties, including the concentration of each components, pH, temperature, etc. In this article, we focus on seven dominant experimental parameters, i.e. the concentration of the seven components, comprising of AM, SA, NaCl, MBA, APS, EDTA-Ca and TEMED. The concentration of those seven components are denoted as variables (features) $X=\left\{x_{1},\;x_{2},x_{3},x_{4},x_{5},x_{6},x_{7}\right\}$ and imported into the dataset (Table S1).

For the practical application of materials, all aspects of material properties need to meet certain requirements. In the case of wearable sensors, materials should possess higher strain sensitivity and elongation in a wide range of strain to accommodate large scale deformations [40]. Furthermore, hydrogels should exhibit not only high resilience and accurate strain detection at relatively low voltages, but also high fracture energy, low resistivity and minimal hysteresis [41]. Herein, we focus on five properties of the hydrogels, including strain sensitivity, elongation, fracture energy, hysteresis and resistivity. To obtain hydrogels with best properties, we introduce machine learning methodology to optimize the experimental parameters. Those five properties are normalized by Equation (6) (Experimental section), linearly weighted (Equation (7), Experimental section) and the weight of each target property is computed using the entropy weight method provided by SPSSPRO. Then the overall material properties Y (label) can be obtained by Equation 8 (Experimental section). The smaller the Y value, the better the properties of the gel.

**Figure 2.**
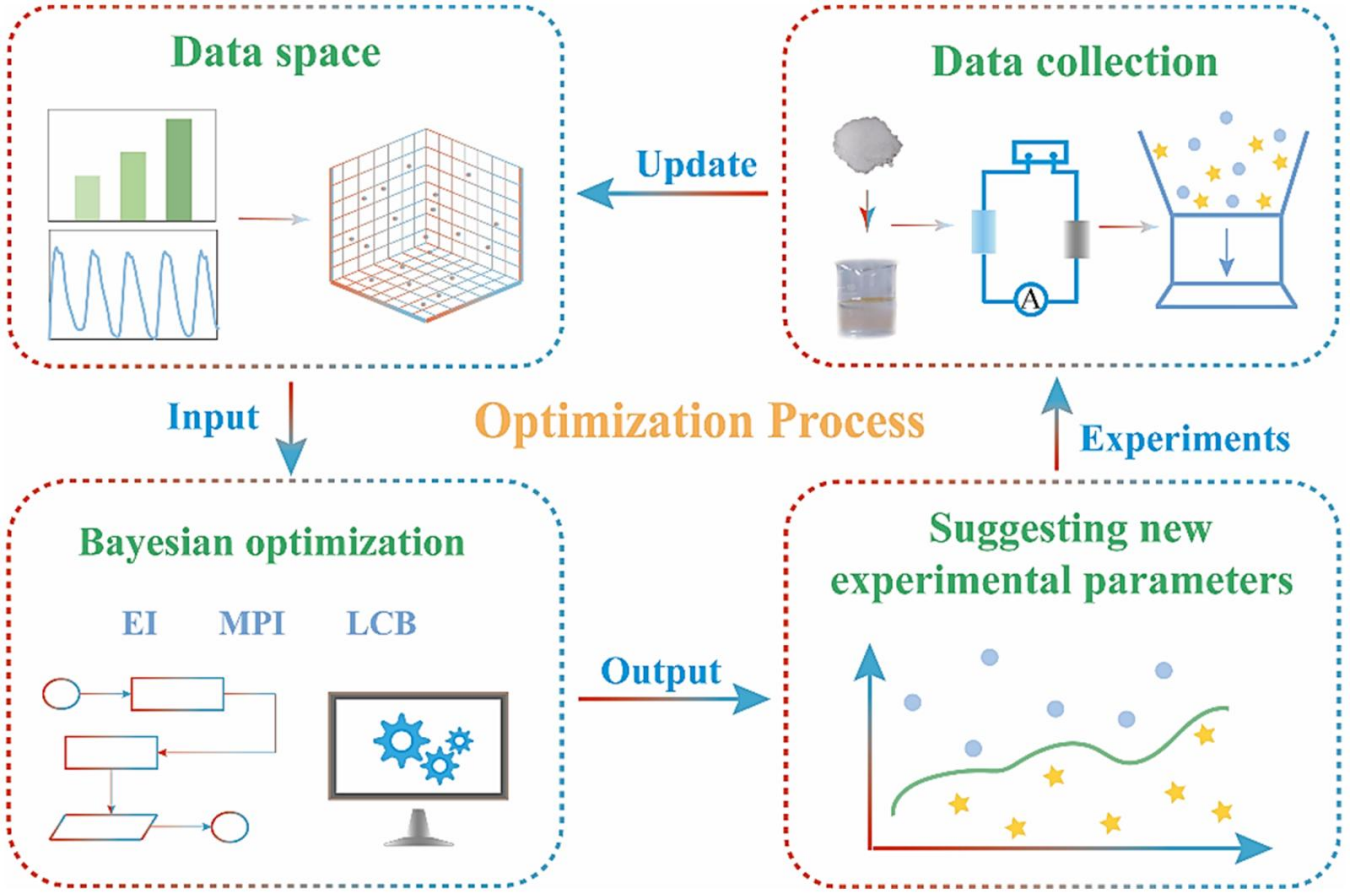
Workflow chart of Bayesian optimization process for optimizing dual network hydrogels.

Figure 2 illustrates the optimization process of hydrogels, showing the learning methodology to lead fabrication process of hydrogels. Prior to optimization, the initial data are utilized to construct an initial data set (experimental parameters as features and *Y* value as label) as the ‘0’ iteration. In each iteration of the Bayesian optimization, three acquisition functions are used, including the EI, MPI and LCB. These acquisition functions play a crucial role in the Bayesian optimization loop, iteratively guiding the exploration of the search space to efficiently locate the global optimum of the unknown objective function. Different sampling strategies strike a balance between exploration and exploitation, facilitating the efficient search for the global optimum. After the dataset is imported to the algorithm, the three acquisition functions respectively derive the possible position X of the component with the best properties in the parameter space based on the existing data set. We fabricate new samples according to the recommended parameters from the machine learning algorithms. Then the sample properties (*y* values) were tested and imported as new data for the new iteration. Iterate this process can expand the dataset continuously and improve the accuracy of prediction.

**Figure 3.**
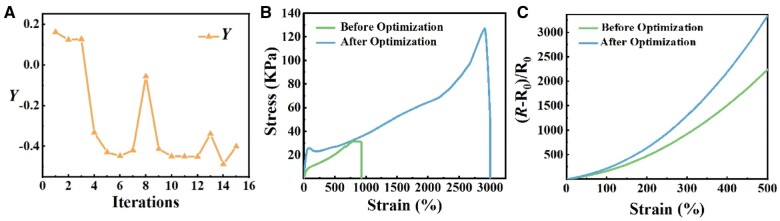
The *Y* value change curve during the optimization process (**A**). A comparison of stress-strain (**B**) and GF curves (**C**) before and after optimization.

Figure 3A presents the overall best properties of hydrogels from each iteration. At the beginning of the optimization (the first three iterations), the *Y* value is relatively stable. At iteration 4 and 5, the *Y* value dramatically decreases. Then the *Y* value fluctuates around −0.45 and reaches the lowest value at iteration 14. Here, we choose the optimized gel from the 14th iteration for the following experiments. Tables S2 and S3 compare the experimental parameters and the properties of gels before and after optimization. Compared with the hydrogel prepared before optimization, optimized hydrogel has a lower *Y* value, indicating a significant improvement in the comprehensive properties of the material. Therefore, with little knowledge about the relationships among experimental parameters and hydrogel properties, we can further obtain hydrogel materials with better properties through the Bayesian optimization process, which proves the efficiency of Bayesian optimization in practical adjustment of experimental parameters.

Based on the weight of the target properties calculated by the entropy weight method, the optimization process focuses more on improving elongation and strain sensitivity. To intuitively assess the disparity in the properties before and after optimization, we compare the optimal materials from the dataset before and after parameter optimization. Figure 3B shows the stress–strain curve of the hydrogel before and after parameter adjustment. Apparently, the elongation of the material has been improved to a certain extent, and the maximum elongation at break is close to 3000%. Through the comparison of the area under the stress–strain curve, the optimized hydrogel can absorb more energy before breaking, with the fracture energy increasing from 0.042 to 0.43 J. It exhibits notable flexibility characteristics, ensuring that the material has a wide range of applications.

In this article, the hydrogel employs sodium ion as a conductive additive, aligning its conductive principle more closely with the conductivity in living organisms. As shown in Figure 3C, the strain sensitivity of the hydrogel is highly increased after optimization. When the same strain is applied, the optimized material exhibits a greater degree of resistance change, resulting in a significant improvement in the GF (defined as the slope of the straight line fit on the GF curve for each stretch range) in each tensile range. The maximum GF value of the optimized hydrogel reaches 11.41 within the testing strain range. As we only use basic ions as conductive additives, it indicates that the hydrogel has relative high sensitivity.

Herein, as a strain sensor, the optimized hydrogel exhibits relatively high or comparable elongation (2999%), strain sensitivity (11.85) and fracture energy (kJ). But it is worth noting that the hysteresis of the hydrogel unexpectedly increases to 0.47, which is relatively high. There are two main reasons why hysteresis has not improved: the complexity of materials science and the limitations of the machine learning method. Numerous influencing factors play a role in the preparation of hydrogels, with each component exerting unique effects on various properties. The relationships between these factors are intricate and not simply linear. However, machine learning algorithms heavily depend on data. The data we have collected from experiments are limited, causing the algorithm to struggle in making precise predictions or optimizations. Additionally, optimizing a material for one property may come at the expense of another property. These trade-offs or other constraints are challenging for the machine learning algorithms to account for, especially with such a small dataset.

### Applications

**Figure 4.**
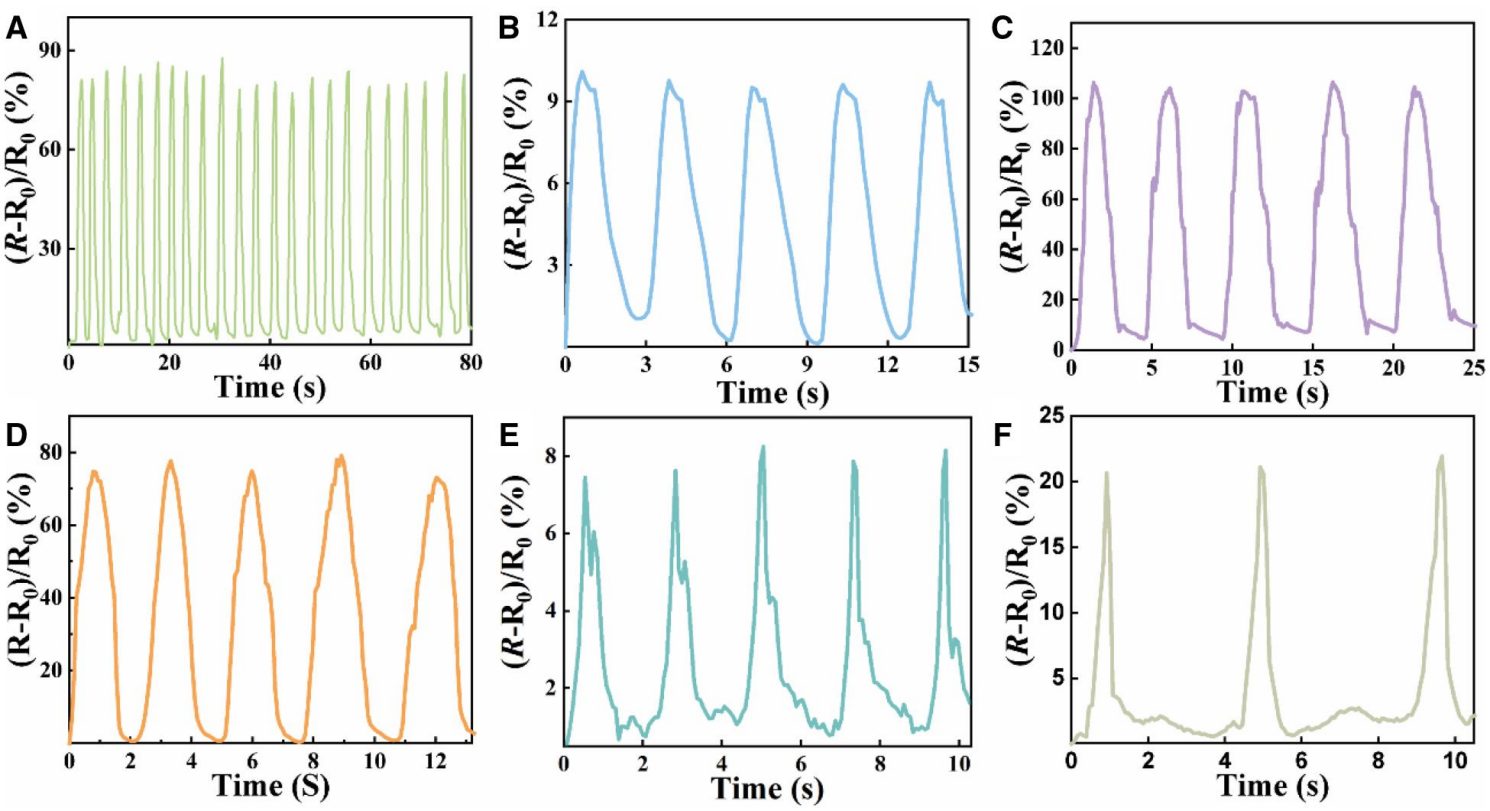
Hydrogel as skin sensor for human motion detection. (**A**) Finger bending. (**B**) Wrist flex. (**C**) Knee movement. (**D**) Elbow movement. (**E**) Vocal cords. (**F**) Throat swallow.

To evaluate the application of hydrogel as a strain sensing material, we create a simple sensor by embedding electrodes inside the hydrogel. When the sensor is placed on various areas of the human body, we can observe and track movements of human skin (Figure 4). Figure 4A illustrates how the rate of resistance change varies with finger movement when the hydrogel is affixed to the finger joint. It can be observed that the sensor responses rapidly during the cyclic finger bending and relaxing process, with repeatable and precise resistance signals. In addition, the signal exhibits excellent repeatability, and the resistance change rate remains robust even after the finger is repeatedly bent and stretched for 23 times, indicating the sensor effectively recognize the bending and stretching motions of the finger and can be used repeatedly. Upon attaching the sensor to the wrist (Figure 4B), knee (Figure 4C) or elbow (Figure 4D), the sensor also exhibits rapid responsiveness. The resistance change rate of the sensor on the wrist is low (∼10%), while the resistance change rate of the sensor on the surface of the knee is the highest (100%). Upon attaching the ionic sensor to the throat, it responds to vocalizations or swallowing by deformation, resulting in corresponding resistance changes (Figure 4E and F). The resistance change rate associated with vocal vibrations is only ∼8%. Thereafter, regardless of large-scale or subtle movement, the ionic sensor prepared by optimized hydrogel materials can effectively monitor the motion and yield consistently stable signals, demonstrating the potential of our hydrogels to be used as strain sensors. Furthermore, the optimized hydrogel also shows low electrical hysteresis, consistent resistance changes at different frequencies, and stable performance in long-term cyclic tests (Supplementary Figure S3), implying that the optimized hydrogel sensor maintains high reliability and precision under varying conditions.

### Machine learning assisting analyzing the impact of experimental parameters on properties

To further explore the correlation between experimental parameters and material properties, we utilize the experimental data as the dataset, employ the machine learning algorithms to analyze how experimental parameters impact hydrogel properties. We collect 213 sets of experimental data after the iteration process of Bayesian optimization, however, we notice that sometimes we fail to obtain hydrogel due to a low degree of gelation (78 sets of negative results) even though we deliberately set the experimental parameter ranges during hydrogel optimization process. We refer to these as ‘cut off’ points. Herein, prior to analyzing and predicting the impact of experimental variables on hydrogel properties, it is essential to exploit a classifier to identify whether the experimental parameters can yield effective hydrogels, thereby aiding in the elimination of experimental parameters that cannot yield hydrogels. This scenario can be regarded as a straightforward two-classification problem, and the machine learning classification algorithms can be employed to establish a classifier to achieve discrimination and analyze the factors contributing to the generation of isolated data points.

**Figure 5.**
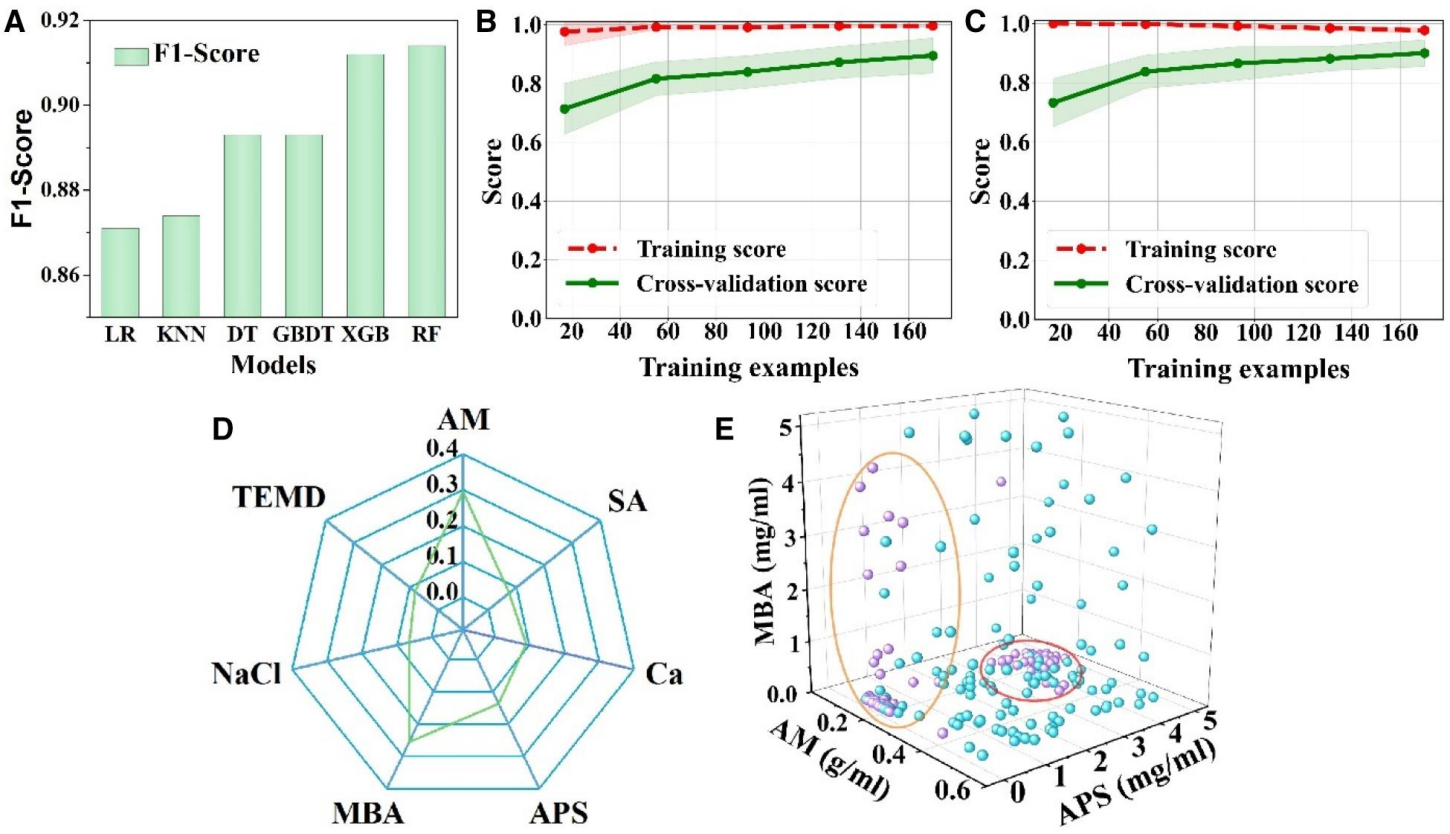
(**A**) *F*1 scores of different classification algorithms. (**B**) Learning curve of RF algorithm. (**C**) Learning curve of XGB algorithm. (**D**) Radar plot depicting the importance of feature in the classification process performed by an RF model. (**E**) The distribution of data points within the three-feature space encompassing MBA, AM and APS.

In this article, data points eligible for obtaining a gel sample are labeled as 1 whereas those for which a gel cannot be obtained are marked as 0. The concentration of seven ingredients listed in Table S4 are utilized as experimental parameters, followed by data normalization. We choose several machine learning algorithms for data training, including logistic regression (LRE), decision tree (DT), and gradient boosting decision tree (GBDT), random forest (RF), extreme gradient boosting (XGB) and *k*-nearest neighbor (KNN) algorithm. After hyperparameter adjustment (refer to Table S5 for hyperparameter details) and training, we evaluate the properties using four quantitative indicators: accuracy, recall, AUC and *F*1-score. Figure 5A and Table S6 demonstrate that XGB and RF exhibit superior accuracy, recall, AUC, and *F*1 score compared to the other four algorithms, namely LRE, KNN, DT and GBDT.

To further assess the fitting properties of the two algorithms, we plotted learning curves using the learning-curve interface in the Scikit-learn library for each algorithm. As illustrated in Figure 5B and C, with an increase in the volume of training data, the cross-validation scores of both algorithms exhibit a tendency to converge to the training scores. But the cross-validation score of RF algorithm converges at a higher level. Consequently, compared with XGB, the RF algorithm exhibits a lower degree of overfitting, making it more suitable for the classification problem presented in this article.

When classifying a model through RF training, the changes in the Gini index at each step can be calculated and accumulated to obtain the influence of each input feature (concentration of each component) on the classification process, i.e. the importance of each input feature (Figure 5D). It indicates that AM has the most pronounced impact (29%), followed by 26% for MBA. The feature importance of other components is 15%, 9%, 8%, 7% and 6% for APS, EDTA-Ca (Ca), TEMD, SA and NaCl, respectively. Therefore, the concentrations of AM, MBA and APS play a more substantial influence on the feasibility of sample preparation. On the other hand, it suggests that, although alginate can form hydrogel without polyacrylamide, inducing gelation in polyacrylamide is comparatively easier than in alginate.

For the gelation of AM, TEMED serves as a catalyst and accelerates the decomposition of APS into free radicals. These free radicals are highly reactive and can initiate the polymerization of AM and MBA. To gain a deeper understanding of the feature importance of AM, MBA and APS, we draw a three-dimensional scatter plot illustrating data points plotted against the concentrations of these three components (Figure 5E). Each component’s concentration is represented along one of the three coordinate axes. The cyan data points represent the experimental parameters capable of producing hydrogels, whereas the purple points signify parameters that cannot generate hydrogels. It can be noted that the purple points cluster into two distinct areas, both characterized by relatively low AM values, but differing in concentrations of APS and MBA. With high APS, there are more free radicals that can be generated in the space to initiate reactions, so the number of polymer chains that can be produced is larger. However, when both AM and MBA concentrations are low (purple points in red oval), it is difficult for locally sparse polymerized AM and/or MBA to form an effective gel network. In contrast, with low APS, low AM but high MBA, MBA can become the dominant component for polymerization, leading to effective gelation. But when MBA values are not high enough (purple points in orange oval), low APS concentration leads to fewer free radicals, significantly reducing the probability of these radicals interacting with AM or MBA molecules. Consequently, AM fails to polymerize effectively, preventing hydrogel formation in this region.

In this hydrogel system, the AM network primarily provides structural support to the hydrogel, while the alginate network is responsible for energy dissipation [39]. Therefore, it is crucial to avoid using experimental parameters that adversely affect these networks.

### Predictive analysis and experimental verification based on regression model

To understand the relationships between experimental parameters and properties, we compared several regression algorithms for data training and selected the best model to predict gel properties. Subsequently, we conducted experiments to validate the feasibility of the model. We employed six algorithms suitable for small datasets to train regression models, including linear regression, polynomial regression, support vector machine, GBDT, XGB and RF. The concentration of the seven components listed in Table S4 were used as experimental parameters, and three crucial property metrics (resistivity, elongation and fracture energy) were used as output variables.

**Figure 6.**
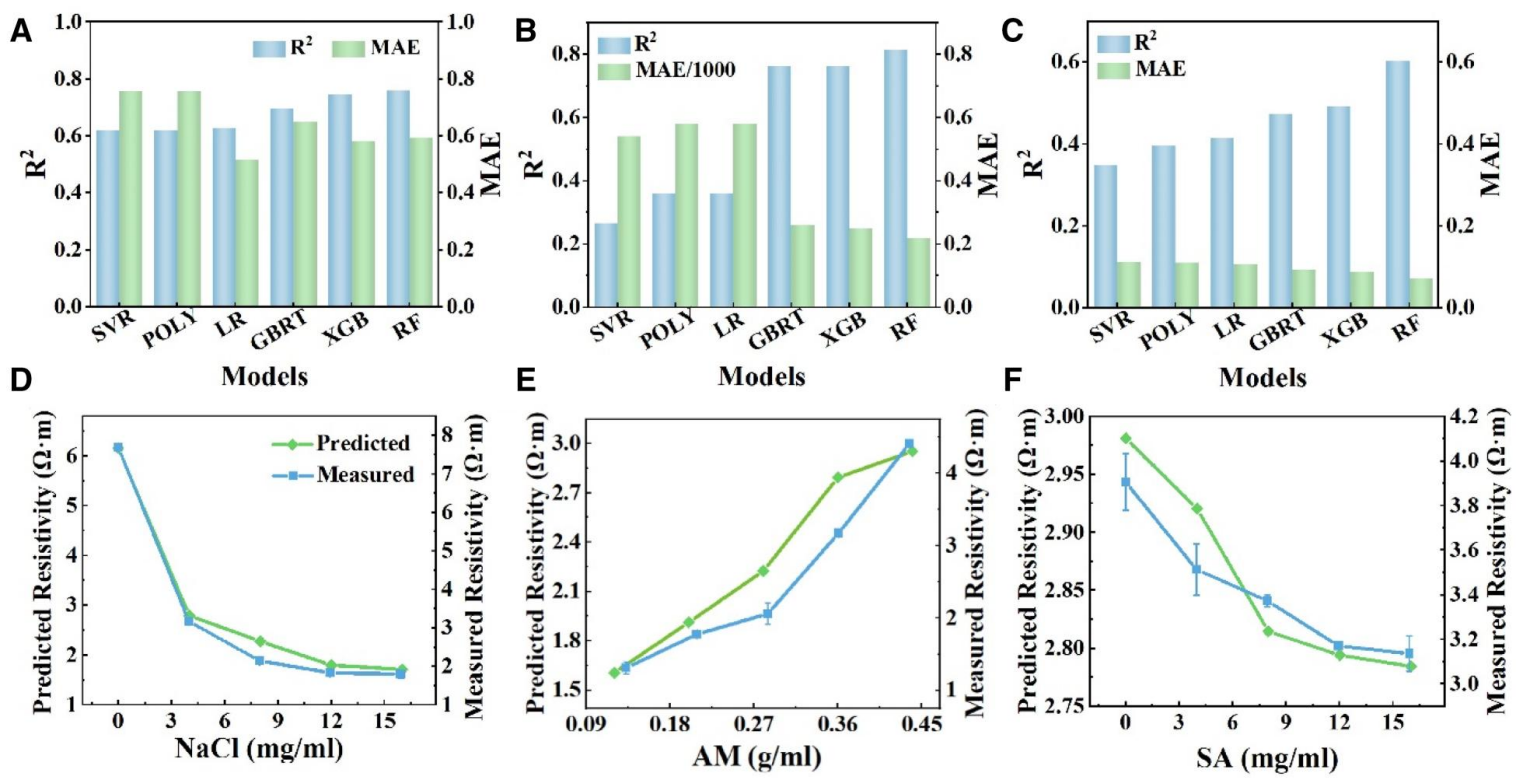
A Comparison of R^2^ and MAE of different regression algorithms when training different models on resistivity (**A**), elongation (**B**) and fracture energy (**C**). A comparison between the predicted and measured resistivity with varied concentration of NaCl (**D**), AM (**E**) and SA (**F**).

Figure 6A–C display the *R* squared (*R*^2^) and mean absolute error (MAE) corresponding to the six algorithm training models, respectively. For detailed numerical values of the four evaluation indicators corresponding to different algorithms during model training, please refer to Tables S7–S9. If the model trained by the algorithm has lower MAE and mean-squared error (MSE), and higher *R*^2^ and explained variance score (EVS), the model can be considered to have better results. Based on this judgment criterion and the fitting of the training data, it can be seen that regardless of the model trained, the RF algorithm consistently exhibits lower MAE and MSE, as well as higher *R*^2^ and EVS, demonstrating better overall properties. Therefore, we utilized the RF algorithm to build models for further prediction and analysis of the relationships between composition and resistivity, elongation and fracture energy.

Upon completing model training, we employ the RF algorithm to forecast and scrutinize the association between specific components and the three-property metrics, demonstrating the practicability of the model. Initially, we employ the algorithm-trained models to investigate how the concentration of three key components (NaCl, AM and alginate) affect hydrogel properties, i.e. the model predicts the resistivity (Supplementary Figure S4), elongation (Supplementary Figure S5) and fracture energy (Supplementary Figure S6) of the hydrogel with varied parameters. Supplementary Figure S4A and B illustrates that an increase in NaCl results in a reduction in resistivity. This phenomenon could be attributed to the heightened concentration of free ions from the increased NaCl, consequently augmenting conductivity. Additionally, as the AM concentration increases, the resistivity increases slightly (Supplementary Figure S4A). This effect is less pronounced when the NaCl concentration is zero, but the change in resistivity becomes more noticeable after adding NaCl. This trend is likely attributed to the introduction of AM, which increases the polymerization of AM and impedes the mobility of free ions. In contrast, change in SA concentration do not alter the resistivity (Supplementary Figure S4B). Supplementary Figure S4C shows that an augmentation in AM content results in a slight increase in resistivity, corresponding to the results in Supplementary Figure S4A. Similarly, with varying SA concentration, an increase in AM concentration leads to higher resistivity (Supplementary Figure S4D). The predictive outcomes depicted in Supplementary Figures S4E and F illustrate that as the concentration of alginate rises, the material’s resistivity slightly decreases, mirroring the patterns observed in Supplementary Figure S4D and B. Similarly, we can use the model to predict and analyze the influence of sample concentration on changes in elongation (Supplementary Figure S5) and fracture energy (Supplementary Figure S6).

To validate the accuracy of the machine learning model predictions, we further conduct experiments to validate the predictions of the model. Figure 6D–F compares the experimental outcomes with the model predictions, with the quality change of NaCl, AM and alginate, respectively. Upon comparison, it is evident that the experimental results are closely aligned with the data predicted by machine learning model, indicating the model can effectively predict the changes in resistivity based on sample quality.

## Conclusion

Hydrogel materials hold promising potential for a wide range of applications. However, the synthesis and preparation of hydrogels typically involves various experimental parameters. Due to the intricate interplay among these parameters, it is challenging to find effective methods for the optimization process. In this study, a strain sensing material using a dual-network hydrogel was developed by combining AM and alginate, targeting application areas like bionics and flexible electronics. Using the component concentration as the experimental parameter and five properties such as strain sensitivity, elongation, fracture energy, hysteresis and resistivity as the evaluation targets, the comprehensive property evaluation index of the material was designed based on the linear weighting method. Taking experimental parameters as input and comprehensive property evaluation indicators as output, a set of experimental plans was constructed using the Bayesian optimization algorithm. Through the iteration of the algorithm, the optimal adjustment of experimental parameters was achieved, we obtain a hydrogel with better strain sensitivity, elongation and fracture energy. The experimental data were further used to train a two-class model using a classification algorithm to effectively sort data. Subsequently, we applied the RF algorithm to establish composition-property mapping models and used them to predict the properties of unknown data point materials, achieving prediction and analysis of the relationship between experimental parameters and properties. Further experimental verification shows that the predicted properties change trend by the model aligns with the properties change trend obtained experimentally, reflecting the practicality of the machine learning model.

## Supplementary Material

rbae109_Supplementary_Data
