# Supplementary material for: Integrating machine learning for the optimization of polyacrylamide/alginate hydrogel
Source: Regen Biomater. 2024 Sep 2;11:rbae109. doi: 10.1093/rb/rbae109 (PMC11422183; doi:10.1093/rb/rbae109)
Supplement: rbae109_Supplementary_Data [file rbae109_supplementary_data.docx]

**Supporting Information**


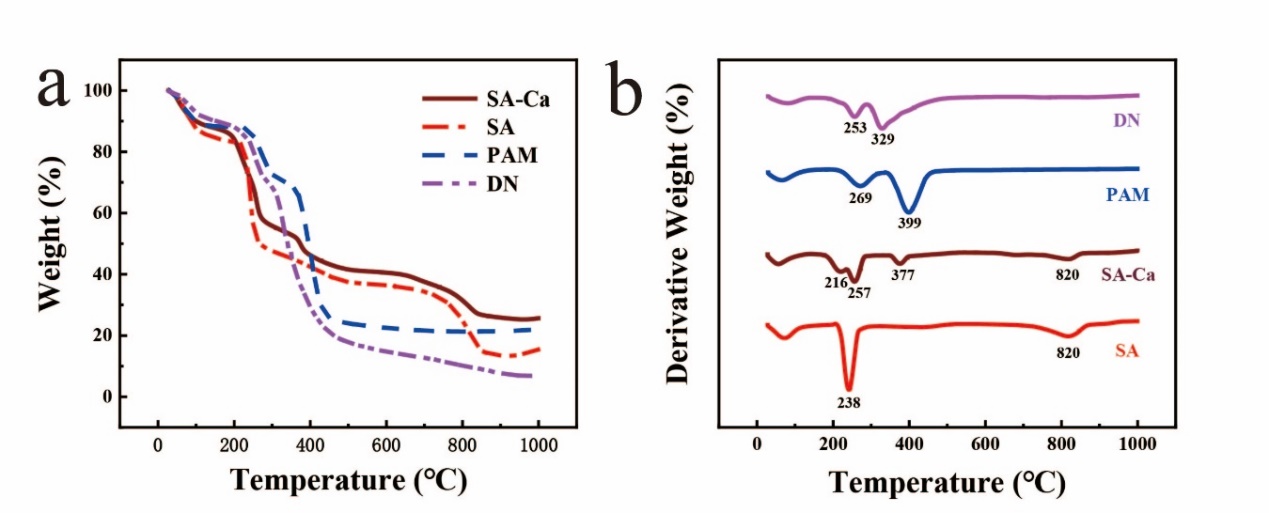


**Figure S1**. Thermogravimetric analysis: (a) TG curves. (b) DTG curves.

**Figure S2**. Infrared absorption spectra of different samples

**
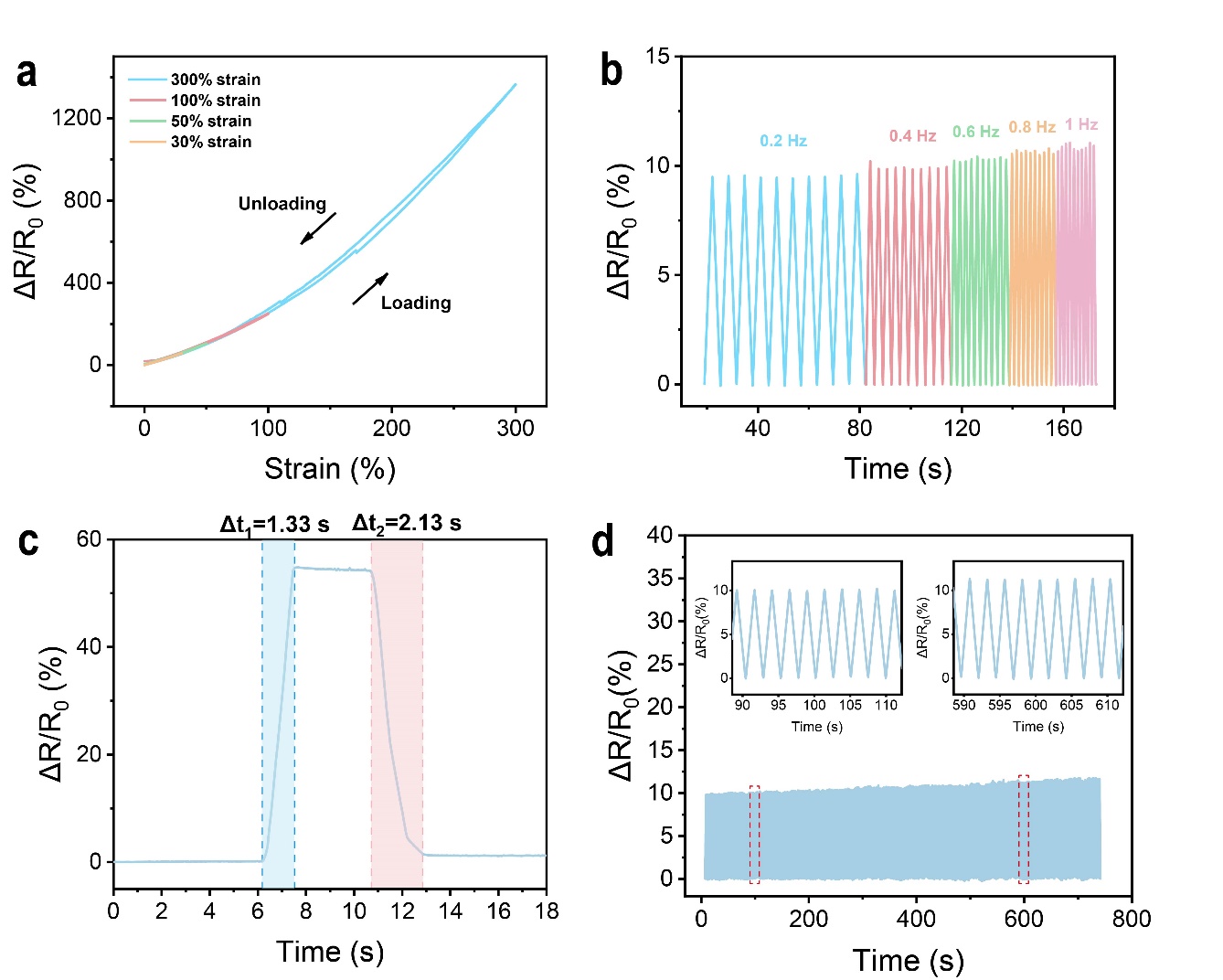
**

**Figure S3.** (a) Electrical hysteresis of the hydrogel sensor measured over a strain range of 0-300%. (b) Resistance changes of the sensor at different frequencies. (c) Response time of the hydrogel sensor. (d) Performance of the sensor under 300 cycles of stretching at 5%.

**Table S1**. Material properties in the initial dataset

| Samples | Elongation  (%) | Strain sensitivity | Fracture energy (J) | Hysteresis | Resistivity  (Ω·m) |
| --- | --- | --- | --- | --- | --- |
| 1 | 248 | 1.35 | 0.05928 | 0.22 | 1.66 |
| 2 | 268 | 0.81 | 0.01848 | 0.20 | 3.23 |
| 3 | 248 | 2.77 | 0.03984 | 0.05 | 3 |
| 4 | 256 | 3.87 | 0.01632 | 0.34 | 4.79 |
| 5 | 206 | 1.29 | 0.03864 | 0.21 | 3.11 |
| 6 | 928 | 8.71 | 0.04224 | 0.27 | 3.34 |
| 7 | 137 | 1.79 | 0.0228 | 1 | 2.59 |
| 8 | 803 | 9.2 | 0.05832 | 0.81 | 0.84 |
| 9 | 203 | 6.97 | 0.01296 | 0.83 | 0.81 |
| 10 | 248 | 2.03 | 0.02664 | 0.40 | 0.84 |
| 11 | 283 | 3.05 | 0.0516 | 0.23 | 2.06 |
| 12 | 184 | 1.88 | 0.02184 | 1 | 1.41 |

**Table S2**. A comparison of component concentrations before and after optimization.

|  | AM  (g/ml) | SA  (mg/ml) | Ca  (mg/ml) | APS  (mg/ml) | MBA  (mg/ml) | NaCL  (mg/ml) | TEMD  (ul/ml) |
| --- | --- | --- | --- | --- | --- | --- | --- |
| Before optimization | 0.20 | 15.64 | 0 | 2.04 | 0.04 | 2.24 | 0.12 |
| After optimization | 0.47 | 2.74 | 12.50 | 1.37 | 0.04 | 2.83 | 0.92 |

**Table S3**. A comparison of hydrogel properties before and after optimization.

|  | Elongation (%) | Strain sensitivity | Fracture energy (J) | Hysteresis | Resistivity (Ω·m) | *Y* |
| --- | --- | --- | --- | --- | --- | --- |
| Before optimization | 928 | 8.71 | 0.04224 | 0.27 | 3.34 | -0.21 |
| After optimization | 2999 | 11.85 | 0.4308 | 0.47 | 4.73 | -0.49 |

**Table S4**. The concentration of each ingredient in the initial data set.

|  | AM  (g/ml) | SA  (mg/ml) | Ca  (mg/ml) | APS  (mg/ml) | MBA  (mg/ml) | NaCL  (mg/ml) | TEMD  (ul/ml) | *Y* |
| --- | --- | --- | --- | --- | --- | --- | --- | --- |
| 1 | 0.4 | 21.92 | 4.72 | 2.68 | 0.68 | 13.52 | 0 | -0.01 |
| 2 | 0.3 | 3.16 | 4.72 | 0.24 | 1.28 | 4.52 | 0.6 | 0.02 |
| 3 | 0.45 | 9.4 | 0 | 5 | 0.68 | 11.24 | 0.6 | -0.06 |
| 4 | 0.5 | 6.24 | 9.4 | 0.24 | 0.68 | 2.24 | 0.84 | 0.00 |
| 5 | 0.45 | 25 | 6.24 | 3.2 | 1.28 | 13.52 | 0.12 | 0.01 |
| 6 | 0.2 | 15.64 | 0 | 2.04 | 0.04 | 2.24 | 0.12 | -0.21 |
| 7 | 0.5 | 12.52 | 1.56 | 3.8 | 1.28 | 11.24 | 0.36 | 0.13 |
| 8 | 0.15 | 25 | 9.4 | 2.68 | 0.04 | 18 | 0.6 | -0.13 |
| 9 | 0.2 | 21.92 | 6.24 | 0.24 | 3.16 | 11.24 | 0.72 | -0.01 |
| 10 | 0.2 | 18.76 | 10.96 | 3.2 | 0.68 | 18 | 0.36 | 0.01 |
| 11 | 0.35 | 12.52 | 12.52 | 4.4 | 0.68 | 6.76 | 0.24 | -0.04 |
| 12 | 0.3 | 25 | 3.16 | 2.04 | 0.68 | 15.76 | 0.72 | 0.11 |

**Table S5**. The hyperparameters utilized in different classification algorithms.

| Model | Hyperparameters |
| --- | --- |
| LRE | penalty = “l1”, multi_class = “auto”, solver = “saga”, C = 0.6, max_iter = 100 |
| KNN | n_neighbors = 13 |
| DT | Max_depth = 2,min_samples_split = 2 |
| XGB | learning_rate = 0.06, n_estimators = 1000, max_depth = 6 |
| GBDT | learning_rate = 0.06, n_estimators = 1000, max_depth = 3 |
| RF | max_depth = 5, min_samples_split = 2, criterion = “entropy” |

**Table S6**. Performance comparison of different classification algorithms.

| Models | accuracy | Recall | AUC |
| --- | --- | --- | --- |
| LRE | 0.881 | 0.866 | 0.923 |
| KNN | 0.870 | 0.884 | 0.924 |
| DT | 0.895 | 0.896 | 0.899 |
| GBDT | 0.901 | 0.888 | 0.955 |
| XGB | 0.919 | 0.907 | 0.960 |
| RF | 0.931 | 0.900 | 0.954 |


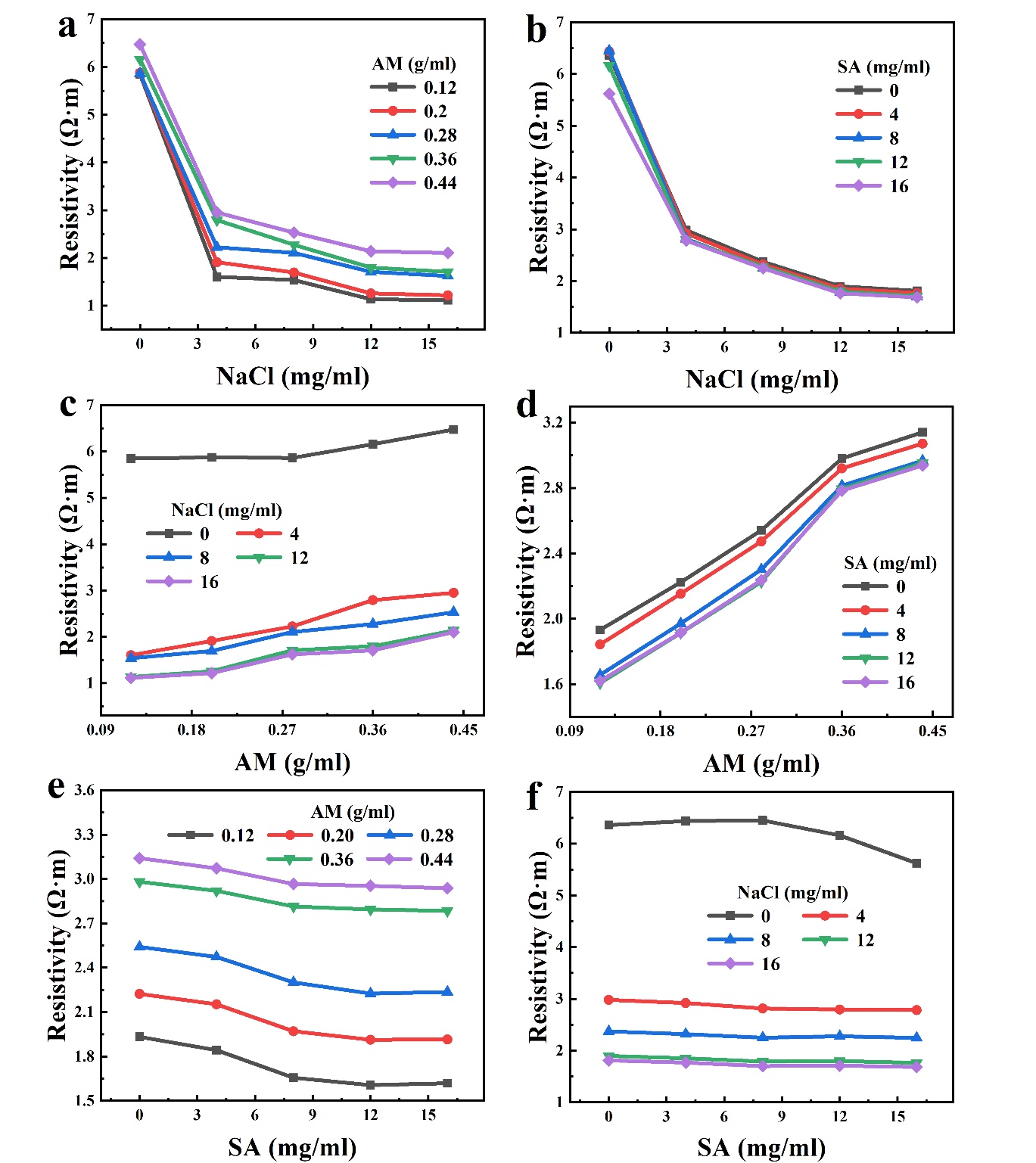


**Figure S4**. Random forest model predicted resistivity as a function of the concentration of different compositions.


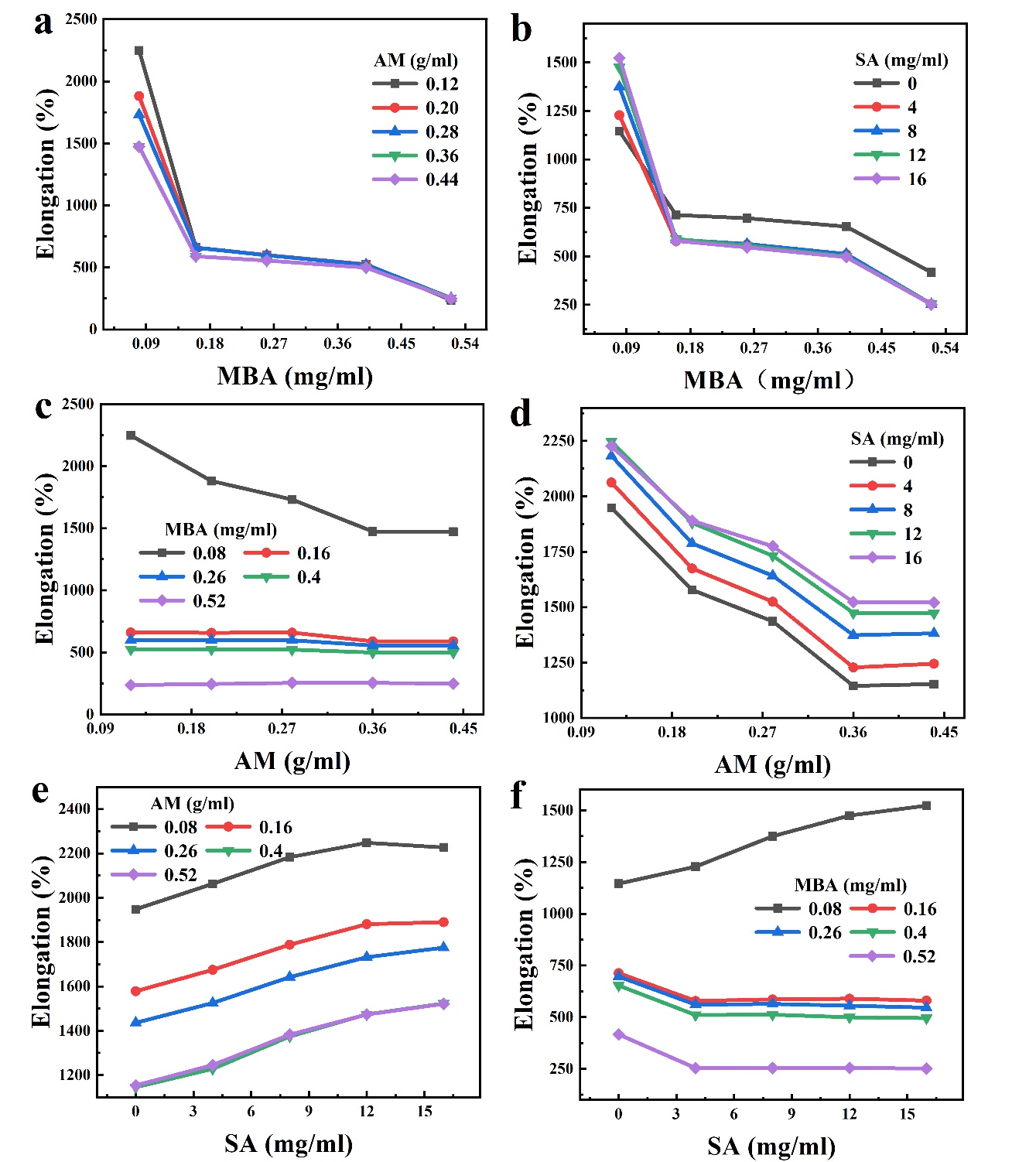


**Figure S5**. Random forest model predicted elongation as a function of the concentration of different compositions.


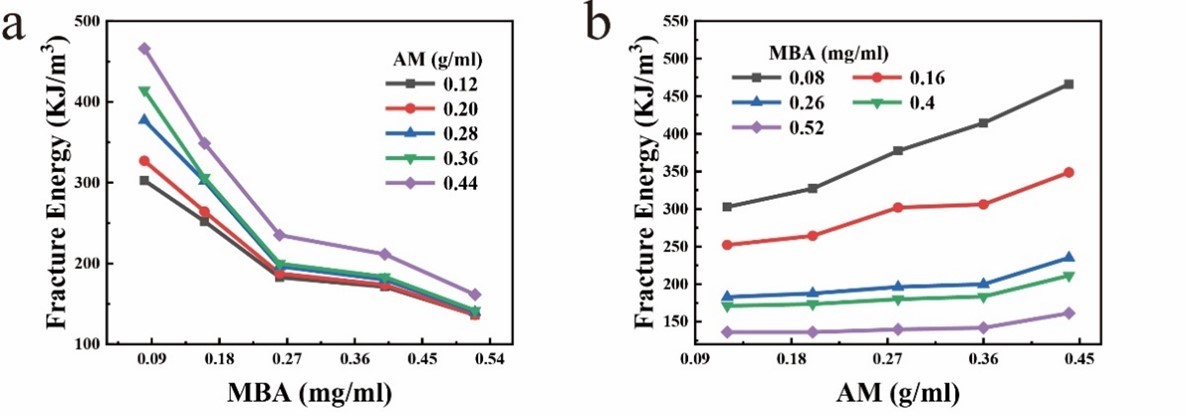


**Figure S6**. Random forest model predicted fracture energy as a function of the concentration of different compositions.

**Table S7.** Performance comparison of different regression algorithms when training different models on resistivity.

|  | RF | GBRT | XGB | SVR | POLY | LR |
| --- | --- | --- | --- | --- | --- | --- |
| EVS | 0.771 | 0.751 | 0.704 | 0.637 | 0.634 | 0.634 |
| R^2^ | 0.759 | 0.744 | 0.696 | 0.627 | 0.619 | 0.619 |
| MAE | 0.594 | 0.581 | 0.649 | 0.517 | 0.756 | 0.756 |
| MSE | 0.786 | 0.819 | 1.123 | 0.804 | 1.094 | 1.094 |

**Table S8.** Performance comparison of different regression algorithms when training different models on elongation.

|  | RF | GBRT | XGB | SVR | POLY | LR |
| --- | --- | --- | --- | --- | --- | --- |
| EVS | 0.818 | 0.767 | 0.769 | 0.280 | 0.377 | 0.377 |
| R^2^ | 0.813 | 0.762 | 0.763 | 0.264 | 0.361 | 0.361 |
| MAE | 219 | 260 | 247 | 540 | 581 | 581 |
| MSE | 129802 | 168565 | 163202 | 476707 | 449981 | 449981 |

**Table S9.** Performance comparison of different regression algorithms when training different models on fracture energy.

|  | RF | GBRT | XGB | SVR | POLY | LR |
| --- | --- | --- | --- | --- | --- | --- |
| EVS | 0.615 | 0.486 | 0.506 | 0.370 | 0.406 | 0.426 |
| R^2^ | 0.603 | 0.472 | 0.491 | 0.348 | 0.396 | 0.414 |
| MAE | 0.073 | 0.092 | 0.089 | 0.113 | 0.110 | 0.106 |
| MSE | 0.014 | 0.020 | 0.020 | 0.023 | 0.021 | 0.020 |
